# Short-Range Vital Signs Sensing Based on EEMD and CWT Using IR-UWB Radar

**DOI:** 10.3390/s16122025

**Published:** 2016-11-30

**Authors:** Xikun Hu, Tian Jin

**Affiliations:** College of Electronic Science and Engineering, National University of Defense Technology, Changsha 410073, China; xikung_hu@126.com

**Keywords:** impulse radio ultra-wideband (IR-UWB) radar, noncontact, short-range, vital signs, ensemble empirical mode decomposition (EEMD), continuous-wavelet transform (CWT)

## Abstract

The radar sensor described realizes healthcare monitoring capable of detecting subject chest-wall movement caused by cardiopulmonary activities and wirelessly estimating the respiration and heartbeat rates of the subject without attaching any devices to the body. Conventional single-tone Doppler radar can only capture Doppler signatures because of a lack of bandwidth information with noncontact sensors. In contrast, we take full advantage of impulse radio ultra-wideband (IR-UWB) radar to achieve low power consumption and convenient portability, with a flexible detection range and desirable accuracy. A noise reduction method based on improved ensemble empirical mode decomposition (EEMD) and a vital sign separation method based on the continuous-wavelet transform (CWT) are proposed jointly to improve the signal-to-noise ratio (SNR) in order to acquire accurate respiration and heartbeat rates. Experimental results illustrate that respiration and heartbeat signals can be extracted accurately under different conditions. This noncontact healthcare sensor system proves the commercial feasibility and considerable accessibility of using compact IR-UWB radar for emerging biomedical applications.

## 1. Introduction

Radar sensors have been widely used in a number of applications since the 1930s [1], from primary vehicle speed measurement to advanced air-defense and marine radars, all of which are usually developed for ranging, targeting, or tracking moving subjects at large distances. Due to its non-invasive and noncontact properties, short-range radar has been an appealing approach in healthcare applications since the 1970s, when the first short-range non-invasive radar for respiration measurement was introduced [2]. Based on the principle of electromagnetic backscattering [3], radar is capable of wirelessly detecting both chest-wall movements caused by respiration and extremely small heart beats. Conventional medical devices like an electrocardiograph (ECG) and respiration belts rely on electrodes alone and an inductive plethysmograph, respectively, which make subjects uncomfortable, and may even worsen the quality of physiological measurements. In long-term monitoring (i.e., for obstructive sleep/coma subjects), an alarm connected to a radar processor can be triggered to either wake the subject or send a message to the subject’s nursing assistants so that they can take measures immediately to avoid possible danger or accidents [4]. In contrast, wearable devices require that the subject be attached to electric poles twisted together with several wires for heartbeat monitoring or a vacuum belt for respiratory monitoring during sleep, which may have a negative impact on sleep quality [5].

Ultra-wideband (UWB) radar is a technology used for transmitting electromagnetic waves spread over a large bandwidth (normally larger than 500 MHz). Typically, most UWB radars transmit via large bandwidth over short pulse periods, usually on the order of a nanosecond, or even a picosecond; we generally refer to this type of UWB signalling as impulse radio UWB (IR-UWB) radar [6,7,8]. This has gained popularity in social and military applications in through-wall imaging, ground penetrating radar, detection of moving targets, and so on owing to its high penetrability and high range resolution [9]. These characteristics make IR-UWB radar attractive for noncontact vital sign detection because it is capable of measuring absolute distance while carrying more vital sign information [10,11,12]. Apart from IR-UWB radar, continuous-wave (CW) radar is a basic type of radar used to detect phase information related to Doppler shift due to a moving chest wall. CW radar falls into three basic subcategories: single-tone, stepped frequency CW (SFCW), and frequency-modulated CW (FMCW) [13]. Each type of radar has its specific advantages. Single-tone CW radar also has a simple system architecture considering high-level chip integration [14], and the phase difference between transmitted and received signals is directly proportional to the target’s motion. On the other hand, FMCW radars can obtain range information, and researchers have also successfully integrated FMCW radar on silicon chips [15]. Moreover, SFCW radars combine some advantages of both single-tone CW radars and FMCW radars and thus have been used successfully in cardio-respiration detection [16]. Li et al. reported a hybrid radar system combining the advantages of single-tone and FMCW radars [17]. With very high-range resolution owing to its wideband nature, IR-UWB radars have been efficiently implemented on silicon [18] and have the potential for realizing accurate detection of respiratory rate and apnea in adults and infants [19]. Above all, each type of radar system can implement the noncontact vital sign detection, depending on the specific application. In this paper, we develop short-range vital sign detection methods using IR-UWB radar based on a fully integrated nano-scale radar transceiver chip, which has a low power consumption, flexible dynamic range and configurable output frequency.

Significant research has been performed to monitor infant respiration using UWB radar, but made no reference to heartbeat detection [20]. Typically, in relaxed human beings, the heart can experience heart displacements of 0.6 mm and respiration displacements are between 12 mm and several centimeters, depending on the person [21]. However, the spectrum of the detected signal contains several harmonics of the breathing signal that can be much stronger than the frequency component of the heartbeat signal [22]. Therefore, it is much more difficult to extract heartbeat signals from complicated radar echo signals. In this paper, the breathing rate and heartbeat frequency are detected remotely and are separated based on a one time-frequency analysis method which combines ensemble empirical mode decomposition (EEMD) with continuous-wavelet transform (CWT). The proposed method can increase the signal-to-noise ratio (SNR) to a certain degree compared with the traditional filtering method [11]. Moreover, experimental results base on proposed method illustrate that respiration and heartbeat signals can be extracted accurately under different conditions. On the other hand, convenience improvements on noncontact monitoring of vital signs through IR-UWB radar systems can be seen from various aspects [20,21,22,23,24,25,26]. In [22] the mathematical modelling of the received waveforms was presented considering the magnitudes of different breathing harmonics and intermodulation, and then non-invasive monitoring of breathing and heartbeat rates was realized using an independent complex generator and sampler, which inevitably made the system heavy. To decrease the weight of the radar system, Khan et al. made it feasible to monitor the vital signs of a non-stationary human using an IR-UWB transceiver chip, but this work did not optimize the complete implementation procedures from the signal mathematical model to the experimental results under practical scenarios [24].

The remainder of this paper is organized as follows: Section 2 presents a mathematical model of vital signs. The signal processing techniques used to detect the respiration and heart rates are presented in Section 3. We describe the proposed IR-UWB sensor system and the experimental setup in Section 4. In Section 5, Experimental results and comparisons are presented. Section 6 concludes this paper.

## 2. Mathematical Model of Vital Signs

By observing the changes in the propagating time delay of the echo signal from a subject, we can detect the range remotely. When the transmitted pulse illuminates a human subject, part of it is reflected back to the radar because of the high reflectivity of the body.

For further digital signal processing, slow-time t is discrete: $t=nT_{s}\;\left(n=0,1,\ldots ,N-1\right)$, where $T_{s}$ is the effective pulse repetition time and N discrete-time sequences are stored after the received signal is sampled.

**Figure 1:**
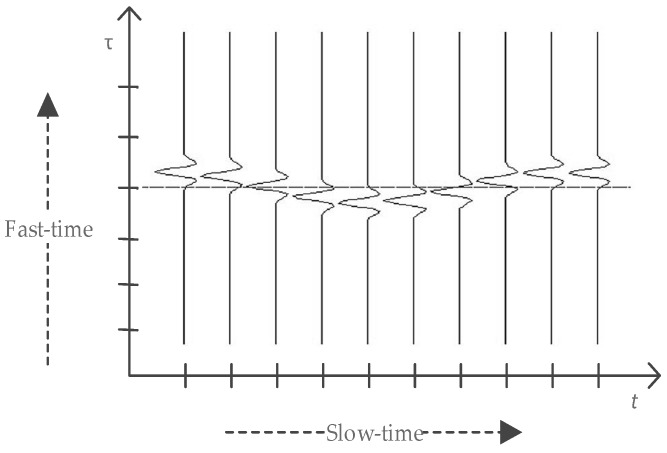
Schematic map of received signal with one respiratory motion and no static targets. t and τ represent the slow-time and fast-time, respectively.

In order to explain this clearly, we show the schematic map of the reflected signal with one respiratory motion and no static targets in Figure 1. The dashed line shows the fast-time bin. The location of the chest changes because of breathing, so the propagation time delay of the reflected signal changes. τ is the propagation fast-time of the electromagnetic wave. If the fast-time is sampled with sampling interval $T_{f}$, and m=0,1,…,M−1 are the fast-time sampling points, the discrete signal can be expressed as an M×N matrix R, the elements of which are [23]:

(1) $$R\left[n,m\right]=\sum_{i}a_{i}s\left(mT_{f}-\tau_{i}\right)+a_{v}s\left(mT_{f}-\tau_{v}\left(nT_{s}\right)\right),$$

where s(t) represents the transmitted signal. $a_{i}$ and $\tau_{i}$ are the amplitude and propagation time delay of static target i in fast-time and $a_{v}$ is the amplitude. $\tau_{v}\left(t\right)$ is the propagation time delay of object reflection in fast-time, and t is the slow-time in which the reflected signal is obtained.

In a static environment, the clutter can be considered as a DC-component in the slow-time direction. From Equation (1) we can see background clutter does not depend on slow-time t. Thus, we can use a basic filter to remove the background clutter; this can be performed by subtracting the mean from the matrix R [23]. Let r(t,τ) represent the received signal; then the signal after clutter suppression can be expressed as:

(2) $$x(t,\tau )=a_{v}s(\tau -\tau_{v}(t))-r_{0}(\tau )=r(t,\tau )-\underset{T\to \infty }{\lim}\frac{1}{T}\int_{0}^{T}r(t,\tau )dt$$

The DC component $r_{0}\left(\tau \right)$ is blocked by subtracting the average of all samples in fast-time.

According to Equation (2) we can obtain the ideal signal without any stationary background as below:

(3) $$y(t,\tau )=a_{v}s(\tau -\tau_{v}(t))=a_{v}s(\tau -\tau_{0}-\tau_{r}\sin(2\pi f_{r}t)-\tau_{h}\sin(2\pi f_{h}t))$$

In order to estimate the respiratory frequency $f_{r}$ and heartbeat frequency $f_{h}$, the Fourier transform of Equation (3) is performed in slow-time:

(4) $$Y(f,\tau )=\int_{-\infty }^{+\infty }Y(f,v)e^{j2\pi ft}dv,$$

After simplifying using the Bessel series [21], the spectrum in slow-time is expressed as below:

(5) $$Y(f,\tau )=a_{v}\sum_{k=-\infty }^{+\infty }\sum_{l=-\infty }^{+\infty }G_{kl}\delta (f-kf_{r}-lf_{h})$$

We can observe from Equation (5) that the spectrum of the signal in the slow-time index is a discrete function which consists of the respiratory rate $f_{r}$, heartbeat rate $f_{h}$ and a train of harmonics. The amplitude $G_{kl}$ is related to the fast-time, and it controls the amplitude of each intermodulation product for a frequency of $f=kf_{r}+lf_{h}$.

## 3. Detection Algorithm

To address the problems of interference from the environment, Wang et al. proposed a combined method in [27], consisting of several general methods to extract periodic signals from noise and clutter, and they also concluded that the performance improvement is not satisfied by using only one method.

**Figure 2:**
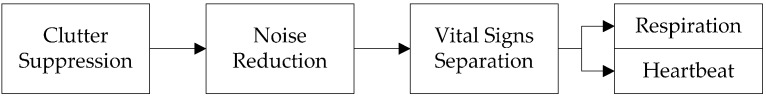
Flowchart of the proposed detection method.

In this section, we propose and elaborate on a detection method combining noise reduction based on EEMD with a separation method based on the CWT. In noncontact vital sign detection, higher SNR improvement is required. Therefore, EEMD is introduced to improve the SNR; after denoising, a separation algorithm based on CWT is used to extract weak heartbeat signals from echo signals. Figure 2 shows the signal processing block diagram.

**Figure 3:**
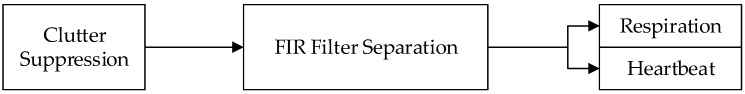
Traditional FIR filtering method.

Generally, a finite impulse response (FIR) filter can be used for cancelling noise and passing a given frequency bandwidth. Since the amplitude of respiration is much larger than the amplitude of heartbeats, observing and separating the two events is possible [11]. Therefore, a traditional processing method using a band-pass FIR filter to separate the vital signs is introduced in Figure 3. According to prior knowledge that the normal heartbeat rate varies from 60 to 100 beats/min (about 1.0–1.6 Hz), in order to obtain the heartbeat signal, the frequency window is set to be 0.65–3.0 Hz. To reject out-of-band noise and to obtain the respiration signal, a low-pass elliptic FIR filter is applied. Comparative experiments between the proposed method and the traditional FIR filtering method are presented in Section 5.1.

### 3.1. Clutter Suppression Algorithm

In a static environment, the clutter from the background can be considered as DC component and removed by subtracting the mean from the matrix R [n,m] in Equation (1) in both rows and columns. To suppress the background of a stationary target and the antenna crosstalk effect, summing and averaging amplitudes along fast-time range bins identifies the strength of the clutter, and owing to periodic amplitude cancellation, x(t,τ) contains little information about vital signs. This is simplified by referring to $x_{m,n}$, denoting the *n*-th slow-time sample of the *m*-th range bins. In the fast-time domain, the detection range is divided into *M* bins. To obtain the position of the target, the ideal number of range bins is calculated as described below:

(6) $$v=\arg\;\underset{m}{\max}(\sum_{n=1}^{N}{\left(x_{m,n}-\frac{1}{N}\sum_{n=1}^{N}x_{m,n}\right)}^{2}),$$

where n=0,1,…,N−1 represents the number of pulses, and m=0,1,…,M−1 is the number of range bins in fast-time. v denotes the selected vital signs bin between 0 and M−1. Finally, the slow-time signal $x_{v,n}$ is the vital sign signal we require.

### 3.2. Noise Reduction Method Based on Improved EEMD Algorithm

The purpose of the empirical mode decomposition (EMD) procedure is to decompose the time series into a superposition of its intrinsic sub-signals (mode function) with well-defined instantaneous frequencies, which are called intrinsic mode functions (IMFs):

(7) $$x(t)=\sum_{i=1}^{n}c_{i}+r_{n},$$

where $c_{i},\;i=1,\ldots ,N$ denotes N IMFs and $r_{n}$ denotes the residue. An IMF is a function that satisfies two conditions: (i) the number of extrema and the number of zero crossing must be equal or differ at most by one; (ii) the mean value of the upper and lower envelopes is zero everywhere. Each of the IMFs represents the oscillation mode present in the data set with different time scale properties. The number of extrema in each IMF is decreased as the IMF order increases and the corresponding spectral supports are decreased accordingly [28]. Each IMF is estimated with an iterative process called sifting. The sifting process consists of five major steps as follows [29]:

1. The maxima and minima of signal x(t) are identified.
2. The upper and lower envelops are obtained respectively by interpolating the set of maximal and minimal points using cubic spines.
3. Computing the mean of the two envelops the mean is designated as $m_{1}$ then subtraction of the mean from the original signal yields $h_{1}=x\left(t\right)-m_{1}$, where $h_{1}$ is the first component presenting difference between the signal x(t) and $m_{1}$.
4. Verifying whether or not $h_{1}$ satisfies the conditions for being an IMF. If $h_{1}$ is not the first IMF, treating $h_{1}$ as the original signal x(t), steps 1–3 are repeated to yield mean $m_{11}$ and $h_{11}=x\left(t\right)-m_{11},$ testing whether or not $h_{11}$ satisfies the two conditions for being an IMF again, if $h_{11}$ is not an IMF, steps 1–3 are repeated k times to yield mean $m_{1k}$ and $h_{1k}=x\left(t\right)-m_{1k}$ until $h_{1k}$ satisfies the two conditions. The first IMF $c_{1}=h_{1k}$ is generated.
5. Subtraction of the $c_{1}$ from the original signal to yield $r_{1}=x\left(t\right)-c_{1}$, where $r_{1}$ is the residue, treating $r_{1}$ as the original signal x(t), steps 1–4 are repeated to yield the second IMF $c_{2}$; repeating this step, the rest of the IMFs of the original signal x(t) are generated, this process can be represented by the following formula:
  (8) $$\begin{array}{c} r_{1}-c_{2}=r_{2}\\ r_{2}-c_{3}=r_{3}\\ \vdots \\ r_{n-1}-c_{n}=r_{n} \end{array}$$

**Figure 4:**
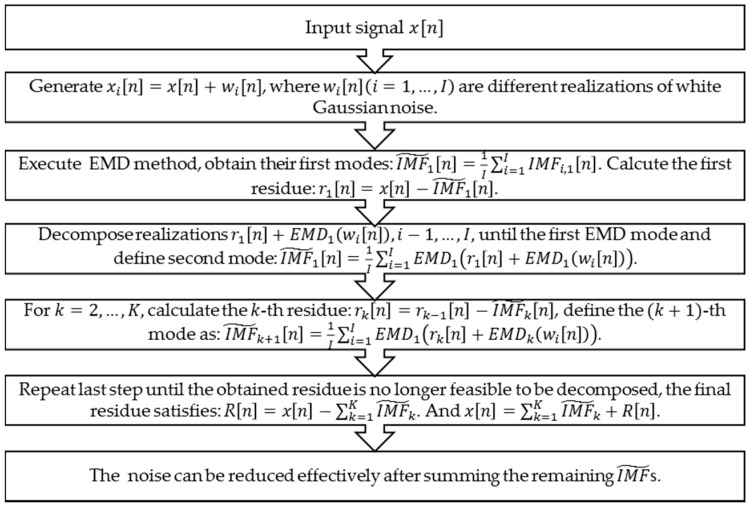
Noise reduction method based on improved EEMD algorithm [28].

In 2011, a variation of the EEMD algorithm was proposed that provides an exact reconstruction of the original signal and a better spectral separation of the modes, with a lower computational cost [30]. Regarding EEMD [31], it defines the “true” IMF components (notated as $\tilde{IMF}$ henceforth) as the means of the corresponding IMFs obtained via EMD over an ensemble of trials generated by adding different realizations of white noise of finite variance to the vital sign signal. Taking full advantage of the IMF components, the improved method used to reduce noise based on the improved EEMD algorithm is shown in Figure 4.

After denoising, the echo signal $x\left[n\right]=x_{v,n}$ obtained by Equation (6) can be rewritten for the *n*-th slow-time:

(9) $$x[n]=\sum_{k=1}^{K}{\tilde{IMF}}_{k},$$

without the residue; on the other hand, we can choose several (not all) of the $\tilde{IMF}$s from k=1,…,K to reconstruct out ideal echo signal.

### 3.3. Separation Method Based on the Continuous-Wavelet Transform

First, we introduce the signal analysis methods, from the frequency analysis method of Fourier transform (FT) to time-frequency methods like the short time Fourier transform (STFT) and CWT, all of which are variations of the FT. Like the FT, the CWT uses inner products to measure the similarity between a signal and an analysis function. In the FT, the analyzing functions are complex exponentials $e^{-j\omega t}$. The resulting transform is a function of a single variable, ω. In the STFT, the analysis functions are windowed complex exponentials, $w\left(t\right)e^{j\omega t}$, and the result is a function of two variables. The STFT coefficients, F(ω,τ), represent the match between the signal and a sinusoid with angular frequency ω in an interval of a specified length centered at τ. In the CWT, the analysis function is a wavelet, *ψ*. The CWT compares the signal to shifted and compressed or stretched versions of a wavelet. Stretching or compressing a function is collectively referred to as dilation or scaling and corresponds to the physical notion of scale. By comparing the signal to the wavelet at various scales and positions, we obtain a function of two variables. If the wavelet is complex-valued, the CWT is a complex-valued function of scale and position. If the signal is real-valued, the CWT is a real-valued function of scale and position. For a scale parameter, a>0, and position, b, the CWT of signal f(t) is:

(10) $$C(a,b;f(t),\psi (t))=\int_{-\infty }^{\infty }f(t)\frac{1}{\sqrt{a}}\psi^{*}(\frac{t-b}{a})dt,$$

where ∗ denotes the complex conjugate. Not only do the values of scale and position affect the CWT coefficients; the choice of wavelet also affects the values of the coefficients. The parameters a and b account for the scaling parameter and translation parameter of the mother wavelet respectively. Scaling and shifting of the mother wavelet produces son wavelets. The scaling factor controls the frequency of the son wavelets; the higher the scale, the lower is the frequency and vice versa. Wavelet coefficients are calculated from the convolution of son wavelets and the signal [32].

After denoising, it is very difficult to extract a heartbeat signal from an echo signal owing to the overlap of dominated respiration amplitude. CWT is used because of their ability to find the energy of the desired frequency interval. Wavelet provides excellent time resolution for rapid events such as heartbeats and good frequency resolution for slower events such as respiration.

The Morlet wavelet is chosen as the mother wavelet to detect the frequencies in received denoised signals and then estimate the amplitude at each detected frequency. The Morlet wavelet is the most frequently used in practice because its simple numerical implementation and because the vanishing of the third-order differentiation of its phase can also simplify the computation [33]. The wavelet transform of a signal f(x) with respect to a mother wavelet g(t) is:

(11) $$S(\tau ,a,f(t),\psi (t))=\frac{1}{2\pi }\sqrt{a}\int F(w)G(aw)e^{iw\tau }dw,$$

where F(w) is the Fourier transform of the signal, a>0 is a wavelet scale parameter, and i is the imaginary unit. τ is a translation parameter and $G^{*}\left(w\right)$ is the complex conjugate of the Fourier transform of g(t). Given the spectrum of an ideal vital sign signal f(x), namely Y(f,τ) in Equation (5), the simplified expression is:

(12) $$F(w)=2\pi \sum_{k=-\infty }^{+\infty }\sum_{l=-\infty }^{+\infty }G_{kl}\delta (f-kf_{r}-lf_{h}).$$

The Morlet wavelet transform of f(x) is:

(13) $$\begin{array}{cc} S(\tau ,a) & =\sqrt{a}f(\tau )\sum_{k=-\infty }^{+\infty }\sum_{l=-\infty }^{+\infty }G_{kl}G_{M}(a(kf_{r}+lf_{h}))\\ & =\sqrt{a}f(\tau )\sum_{k=-\infty }^{+\infty }\sum_{l=-\infty }^{+\infty }G_{kl}G_{M}(a_{r}f_{r}+a_{h}f_{h}) \end{array}$$

The explicit expression of a Morlet wavelet in time domain and frequency domain, respectively, are:

(14) $$g(t)=\frac{1}{\sqrt{\pi f_{b}}}e^{j2\pi f_{0}t}e^{-\frac{t^{2}}{f_{b}}}$$

(15) $$G(f)=\frac{1}{\sqrt{\pi f_{b}}}e^{-\pi^{2}f_{b}{\left(f-f_{0}\right)}^{2}}$$

where $f_{b}$ is the bandwidth parameter of the mother wavelet, $f_{0}$ is the center frequency of the wavelet, and t is the time. The center frequency depicts the ensemble characteristics of the wavelet, and the bandwidth parameter controls the shape of the wavelet [34]. The Fourier transform of the mother wavelet function corresponding to scale a is given by following equations:

(16) $$g_{M}(t)=\sqrt{\frac{1}{a\delta }}g(\frac{t}{a\delta })$$

(17) $$G_{M}(f)=\sqrt{\frac{\delta a}{\pi f_{b}}}e^{-\pi^{2}f_{b}{\left(a\delta f-f_{0}\right)}^{2}}$$

where δ is the sampling period. The value of the $G_{a}\left(f\right)$ will reaches a peak when $a\delta f=f_{0}$. The value of the localized frequency component of the signal can be retrieved as follows:

(18) $$\left\{ \begin{array}{l} f_{r}=\frac{f_{0}}{a_{r}\delta }=\frac{f_{0}f_{s}}{a_{r}}\\ f_{h}=\frac{f_{0}}{a_{h}\delta }=\frac{f_{0}f_{s}}{a_{h}} \end{array}\right.$$

where $f_{s}$ denotes the sampling frequency. From Equation (18), we can conclude that the $f_{r}$ and $f_{r}$ correspond to each specific wavelet scale $a_{r}$ and $a_{h}$, with known $f_{0}$ and $f_{s}$.

**Figure 5:**
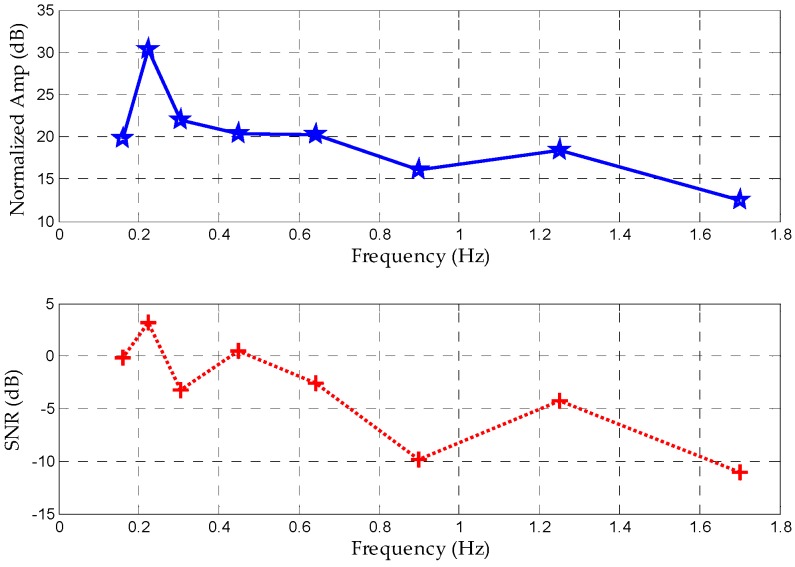
Normalized amplitudes and SNRs of different scales in terms of corresponding peak frequency based on prior knowledge.

To simplify the computation, we usually discretize the wavelet scale a, using the dyadic representation $a=2^{m}$ which is also called the dyadic wavelet transform (DWT). The sampling frequency of the received signal is 65 Hz, roughly equal to the dyadic 6, represented as $f_{s}=2^{6}$. On the other hand, we select the dyadic representation of the center frequency as $f_{0}=2^{8}$, which is an empirical value; this is usually chosen to be two orders of magnitude larger than the sampling frequency. The dyadic representation of wavelet scale of respiration signal can be referred to as:

(19) $$a_{r}=\frac{2^{8}\times 2^{6}}{2^{-2}}=2^{16}$$

which is displayed on a wavelet scale of 16. Usually, this is an estimated empirical value. Using the same estimation method, we can select the preferred coefficient of 12 as the heartbeat wavelet scale. Therefore, there is no need to compute the wavelet transform at all scales. A rough approximation of the scales $a_{r}$ and $a_{h}$ may be read from the Fourier transform of the wavelet signal, or derived from a prior knowledge. As showed in Figure 5, the scale of 16 represents the ideal frequency of 0.22 Hz with an SNR of 3.15 dB and an amplitude of 30.26 dB below 0.65 Hz. Between the heartbeat filtering bands from 1.0 to 3.0 Hz, the peak frequency of 1.25 Hz on a scale of 12 can be filtered with an amplitude of 18.41 dB. If we select these two scales as the ideal CWT scales for separating the vital signs, estimated accuracy reaches a relatively high level.

**Figure 6:**
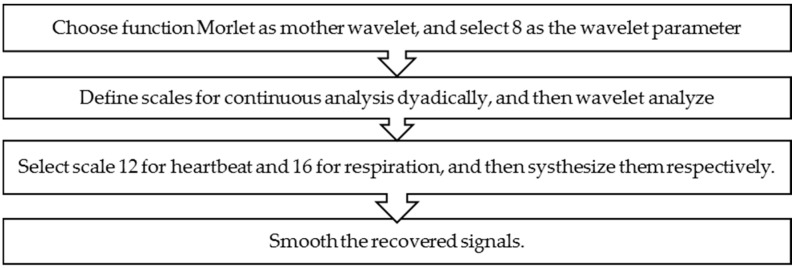
Flowchart of the separation method using the Morlet CWT.

The application of the Morlet wavelet decomposes the signal into a series of components and for each decomposition levels, the coefficients can be either set to zero or reduced in magnitude, so that a particular feature of the signal is affected upon reconstruction. The high-frequency (HF) components are used for heartbeat signal recovery and the low-frequency (LF) ones for respiration recovery. Finally, a moving average filter is applied to each signal; thus, the heartbeat and respiration signals are reconstructed. The signal processing for recovering ideal signals can be summarized as in Figure 6.

## 4. Radar System and Experimental Setup

### 4.1. Radar System

**Figure 7:**
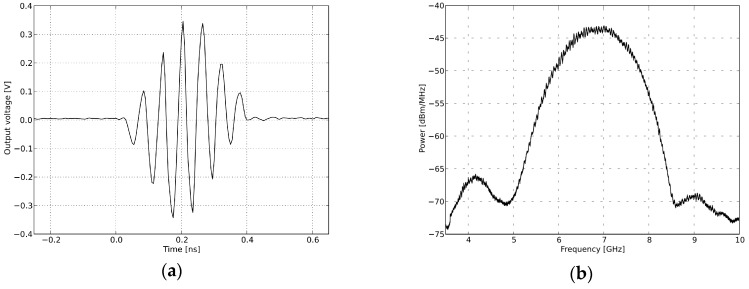
Transmitted signal in the time domain and frequency domain. (**a**) Pulse generator time domain output; (**b**) pulse generator output spectra.

**Figure 8:**
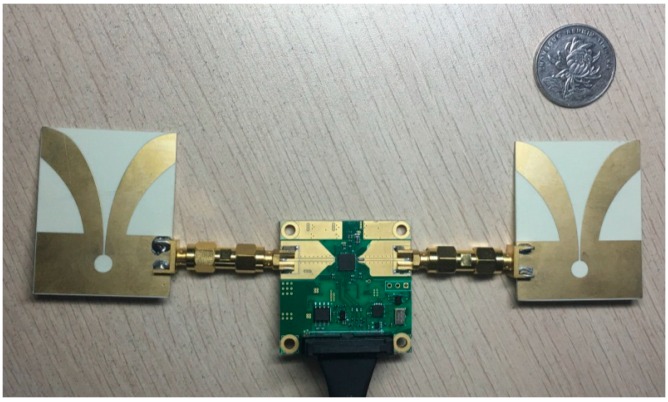
IR-UWB radar system.

The design of the sensor is based on a fully integrated nano-scale radar transceiver chip, which has a low power consumption, flexible dynamic range and configurable output frequency [35]. The pulse generator used to transmit a narrow Gaussian pulse signal like the one depicted in Figure 7a, with a 2.3-GHz output frequency band as showed in Figure 7b. After enhancement by the power amplifier, the signal is emitted by Vivaldi transmitting antennas as shown in Figure 8. In the receiver, the reflected echo is first received by the receiver antennas and then magnified by a low-noise amplifier (LNA). Next, the signal containing vital sign information is sampled by a high-speed sampler. Finally, the digital signal is transferred to a MATLAB processor on a computer with a slow-time frequency of about 65 Hz [36].

### 4.2. Experimental Setup

**Figure 9:**
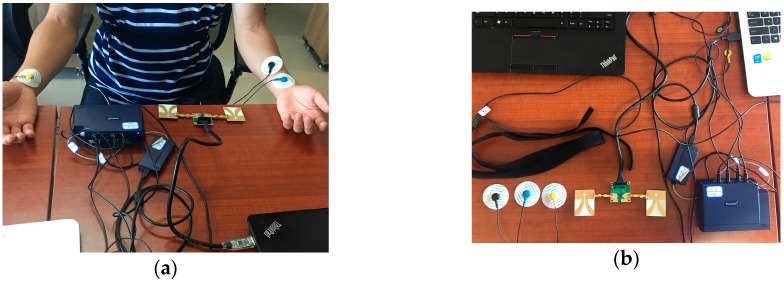
Experimental setup. (**a**) Experimental scenario; (**b**) the radar sensor and DynaMap Suite.

Figure 9 shows the experimental setup of the radar used for measurement. The volunteer is a 22-year-old healthy male sitting in a chair and breathing regularly while medical devices simultaneously monitored him, at a distance of around 0.3 m as shown in Figure 9a. The specifications for the measurement are given in Table 1. The radar has an average output power of 55 μW, which complies with Federal Communications Commission (FCC) regulations for consumer electronics in the band of 2.3 GHz at the center frequency of 6.8 GHz (FCC 1993). This means that this radar system does not harm subjects when operating. In addition, all experimental processes and configurations were undertaken with the consent of the subjects before beginning, and all subjects agreed to cooperate with us to ensure smooth execution of the experiments.

In order to evaluate the performance of the radar system, we applied a comprehensive sports medicine tool (DynaMap Suite–SA7925, Thought Technology Ltd., Montreal West, QC, Canada) in Figure 9b, as the measurement reference that can monitor several medical indices consisting of ECG, HR, IBI, and respiration, etc., using BioGraph Infiniti Software (Thought Technology Ltd., Montreal West, QC, Canada) to capture and export the respiratory data and ECG data.

## 5. Results

### 5.1. SNR Comparison of FIR Filter and Proposed Method

**Figure 10:**
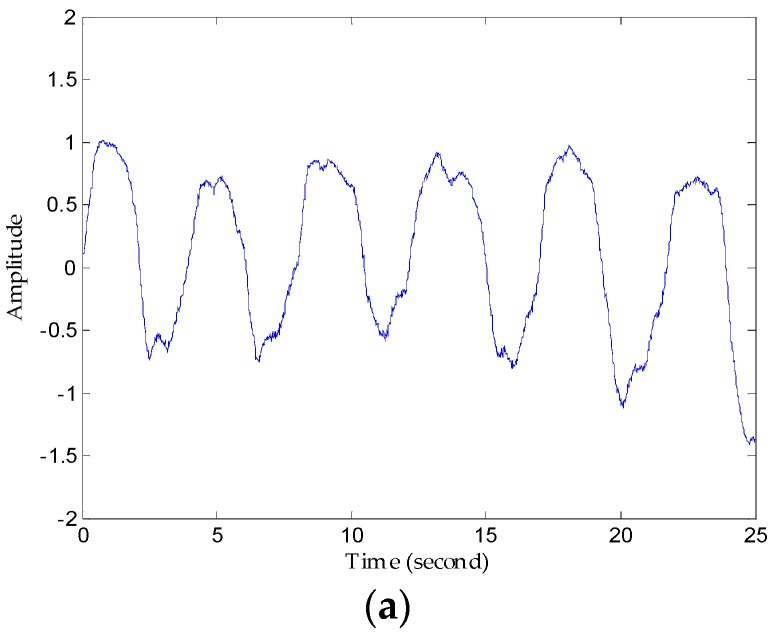

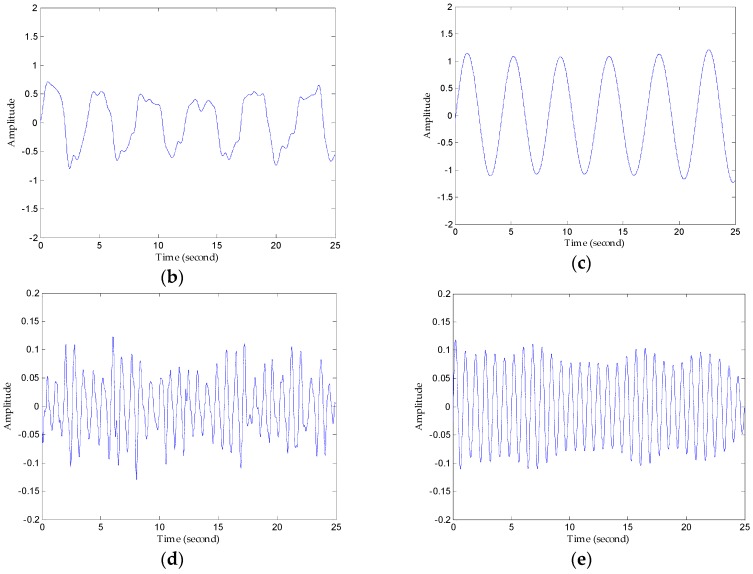
Comparison of results using the FIR filter and the proposed method. (**a**) The original waveforms obtained after clutter suppression; (**b**) respiration waveforms obtained via the FIR band-pass filter; (**c**) respiration waveforms obtained via the proposed method; (**d**) heartbeat waveforms obtained via the FIR band-pass filter; (**e**) heartbeat waveforms obtained via the proposed method.

Figure 10a refers to the original vital signs obtained after clutter suppression. From the respiration waveforms in Figure 10b,c, estimating the respiration rates is possible since their peaks and valleys are quite obvious, but the respiration signal in Figure 10c has a more legible tendency and more defined signatures than that in Figure 10b in the time domain. On the other hand, owing to lower reflected energy, the heartbeat waveforms in Figure 10d have peak-peak values of just about 0.2 mV, and the waveform obtained using the FIR filter displays an irregular sign without any unambiguous heartbeat tendency, whereas the heartbeat waveforms in the decomposed results are stable and regular in Figure 10e. Above all, the proposed method works better than the traditional FIR method in sensing vital signs.

The SNR criterion depends on the performance and detection precision system required, in other words, prior information can be used to determine a reasonable SNR for recognizing the respiration and heartbeat rate so reference vital signs signals are necessary to determine the unstable SNR standard. For qualitative analysis, the SNR of the vital signs signal is redefined in the frequency domain [37]. If the respiration rate is estimated as the frequency $f_{max}$ of the peak in the frequency spectrum, the SNR is calculated as below:

(20) $$SNR=10\log_{10}\frac{\int_{f_{\max}-B/2}^{f_{\max}+B/2}\left|P_{x}(f)\right|df}{\int_{0}^{\infty }\left|P_{x}(f)\right|df-\int_{f_{\max}-B/2}^{f_{\max}+B/2}\left|P_{x}(f)\right|df}$$

where B=0.016 Hz is the resolution in periodogram estimation, which is determined by the number of FFT point and the sampling frequency we demand. SNR calculations for respiration and heartbeat rates are shown in Table 2. The SNR improvement for respiration detection by the method described in this paper is 7.59 dB; moreover, the SNR improvement for heartbeat signals is 4.82 dB. Higher SNR is of vital importance for radar systems helping to decrease the false alarm probability; this is especially significantly for medical monitoring applications. In addition, higher SNR can contribute to improving detection accuracy in more complex environments, and increasing the detection range, which can help IR-UWB radar to adapt in many different environments and even to different human postures.

### 5.2. Detection Performance of Proposed Method

**Figure 11:**
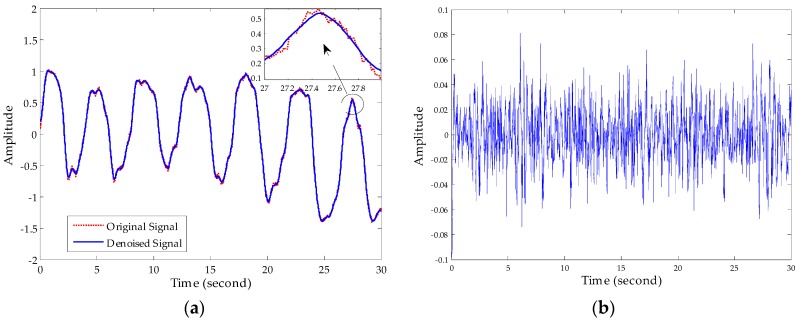
Performance of noise reduction. (**a**) Comparison of original vital sign signal with the denoised signal; (**b**) noise removed after denoising processing.

**Figure 12:**
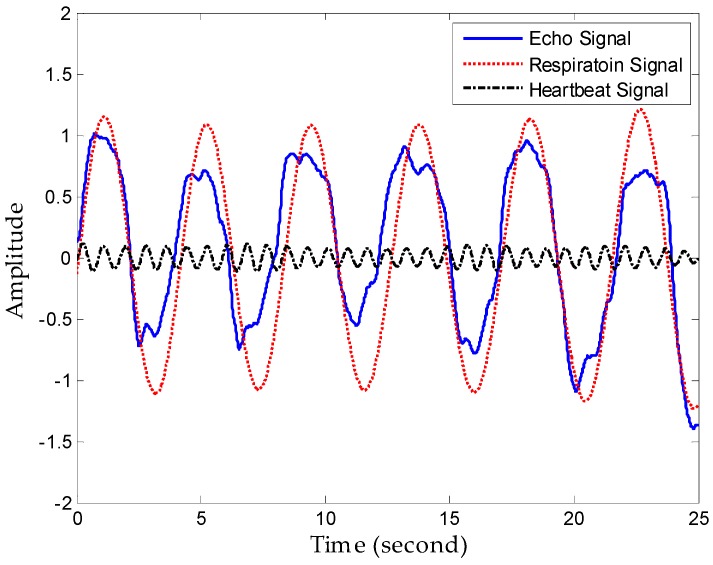
Performance of separation.

**Figure 13:**
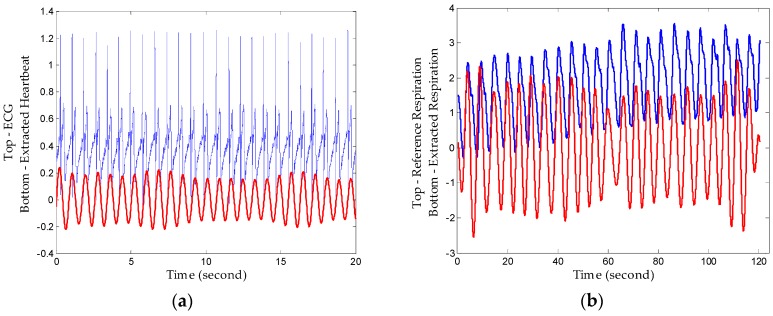
Extracted signals compared with reference signals. (**a**) Extracted heartbeat signal and ECG reference signal; (**b**) extracted respiration signal and respiration reference signal.

First, we denoised the signal and then separated the vital signs using the CWT method. As Figure 11 shows, it can be seen from the enlarged view that the noise attached to the signal has been removed after denosing processing using an algorithm based on EEMD. Then, we used the Morlet wavelet to analyse the denoised signal and chose the scale of 12 to synthesize the respiration signal and 16 scale for the heartbeat signal effectively. The results of signal separation presented in Figure 12, from which we observe that accurate respiration signal waveforms and exact heartbeat signal waveforms can be obtained. Finally, the examples of 20 s of an extracted heartbeat signal and ECG reference signal are compared in Figure 13a, and 120 s of an extracted respiration signal and respiration reference signal are compared in Figure 13b.

The results show that heartbeat signals superimposed on respiration signals can be decomposed with clear peaks corresponding well to the ECG, and decomposed respiration results also show a high consistency with the reference signal. Therefore, the proposed method realizes comparable detection performance to professional medical device with a high conformance to healthcare indices. Additionally, the respiration rate and heartbeat rate can be calculated simply after tracking peaks in the frequency domain or zero-crossings in time domain with low relative error since they have big amplitudes and long duration.

**Figure 14:**
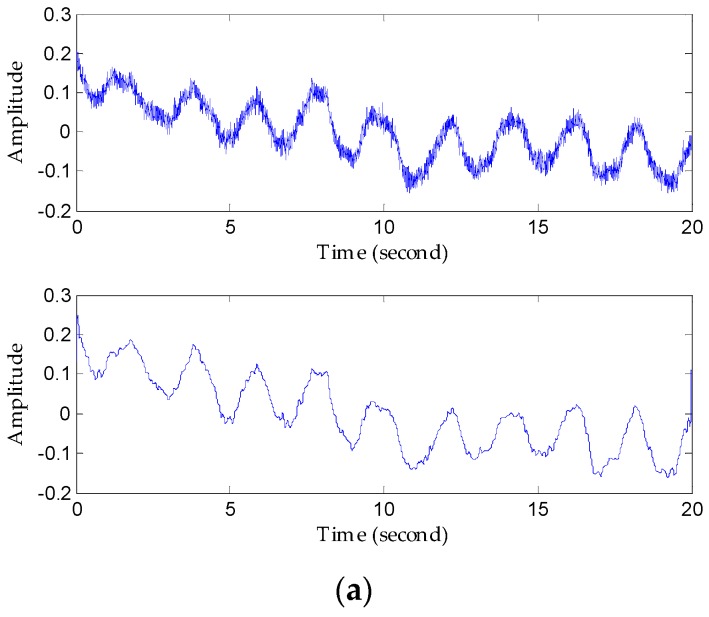

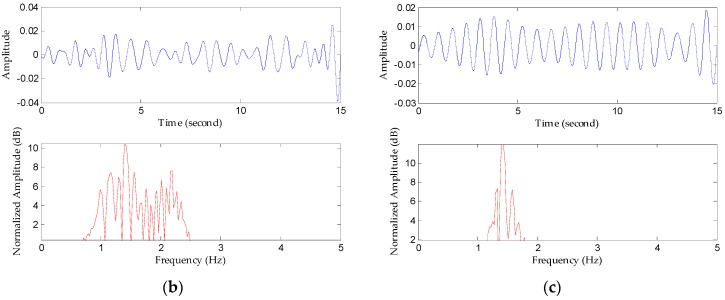
Comparison of results using the FIR filter and the proposed method to recover heartbeat waveforms 5 m away from the detector. (**a**) Original signal waveform and the denoised waveform; (**b**) heartbeat signal waveform extracted using the FIR filter and its spectrum; (**c**) heartbeat waveform extracted using the proposed method and its spectrum.

Next, we focus on evaluating the ability to extract the weak heartbeat when the subject is 5 m away. In facilitating the heartbeat separation, it is obvious that larger-distance detection places a limitation on the detection of such weak signals in Figure 14a; hence it is necessary to denoise to in order to find useful heartbeat signals. Figure 13b presents a chaotic waveform in the time domain and a high side lobe spectrum, which illustrates that the FIR processing in this case failed owing to poor SNR and indistinguishable peaks. On the other hand, the heartbeat waveform obtained using the proposed method has a sine-like tendency, and more apparently, the high resolution in frequency domain in Figure 14c. The reference heartbeat is about 1.42 Hz, which represents ideal detection accuracy.

**Figure 15:**
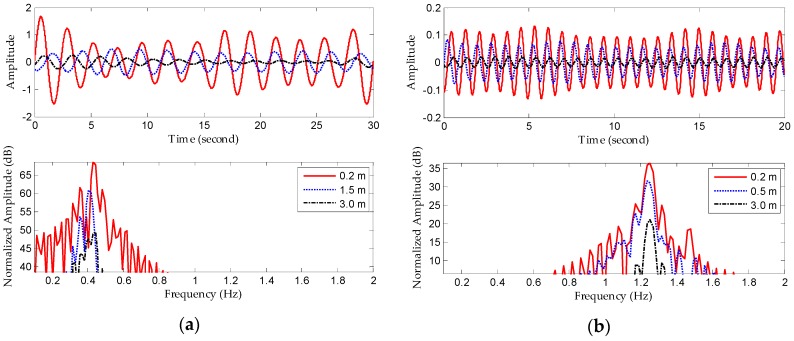
Detection results with different subjects at different distances. (**a**) Extracted respiration signals and frequency spectra; (**b**) extracted heartbeat signals and frequency spectra.

For further validation under various conditions, we pursued experiments in which we examined three subjects (a 13-year-old teenager, a 30-year-old thin woman, and a 56-year-old obese man) sitting at 0.2-, 1.5-, and 3.0-m distances, respectively. These subjects had different radar cross sections (RCS); usually, however, the process of capturing and re-radiating power is very complicated and each subject has the time-varying vital signs. Therefore, there is no need to test all three distances on all three subjects. If we can determine the heartbeat rate of an elderly obese man at the farthest distance, we can be sure that a trial with the young teenager subject would obtain better detection performance, because it is more difficult to penetrate the correspondingly deeper fat distribution. In contrast, testing this with an energetic young person enables accurate measurement of larger changes in movement of the chest. By locating the different range bins in fast-time, we can identify the locations of subjects using power-spectrum density analysis; each original signal at three different locations can then be captured. Figure 15 shows the corresponding processing results after obtaining the respective original echoes, from which the amplitudes of vital signs are seen to decrease with increasing range. In addition, we can estimate the respiration rate and heartbeat rate. Experimental results based on EEMD and CWT demonstrate reliable detection accuracy.

## 6. Conclusions

This paper describes a method for short-range vital sign sensing using IR-UWB radar that only eliminates the trade-off of low power consumption and system complexity versus affordable price, but also carries more range information owing to the benefits of UWB. When radar operates, clutter caused by indoor static objects and antenna crosstalk is common and very serious. In this case, the SNR of the echo signal is so low that it is difficult to extract vital sign signals from complicated background clutter and noise. In order to minimize the signals of interest, several methods were used to improve SNR in this study. The proposed method involves sequential clutter removal, denoising based on EEMD and separation based on CWT. Compared with the traditional FIR filtering method, the SNR of the extracted respiration and heartbeat signals were raised by 7.59 dB and 4.82 dB, respectively. Moreover, experimental results illustrate that respiration and heartbeat signals can be extracted accurately under different conditions.

This system can measure heartbeats based on the proposed method. On this basis, the system can be used in smart home healthcare, which is becoming very popular. As health-monitoring technologies advance further, we envision monitoring people’s vital signs including breathing and heartbeat signals, especially those of sleeping children or the elderly. These patients can use this information to enhance their health-awareness. In addition, the resulting beats can be used to compute emotion-dependent features which can be fed to a machine-learning emotion classifier. These advantages allow us to build machines that enable smart homes that can react to our moods and adjust lighting or music accordingly. Moreover, this also allows filmmakers to benefit from better tools to evaluate user experiences. Advertisers can learn of customers’ reaction immediately. We believe that this will be a trend in the future [38]. In addition, owing to the range resolution and low-frequency penetration of UWB, we can also use it to extend our senses, enabling us to detect vital signs through walls or behind the closed doors.

Non-contact measurement of vital signs using IR-UWB marks an important step towards monitoring accurate respiration and heartbeat rates. However, it has some limitations, which are lited below and left for future work as mentioned above:

1 Sleep monitoring places higher requirements for real-time signal processing. Additionally, the influence of the orientation of a non-stationary human body with changeable sleeping positions must be considered, which is of vital significance for long-term monitoring. Therefore, further work will include an improved algorithm based on the proposed one, enabling it to adjust to non-stationary human subjects [39].
2 To recognize emotions, we must measure minute variations in each individual heartbeat’s length [40]. However, extracting individual heartbeats from radar signals involves multiple challenges. Obtaining such accuracy is particularly difficult in the absence of sharp features that identify the beginning or end of a heartbeat.
3 When faced with a non-metallic wall, a fraction of the radar signal travels into the wall, reflects off objects and humans, and returns to the detector imprinted with the signature of what is inside a closed room. By capturing these reflections, we can estimate vital signs like breathing and heartbeats. However, this is difficult because the signal power after traversing the wall twice (into and out of the room) is reduced by three to five orders of magnitude [41]. Weak heartbeat signals are so weak that using the previous methods cannot extract them accurately.

## Figures and Tables

**Table 1:** Specifications for the measurement.

Parameters	Specifications
Center Frequency	6.8 GHz
Bandwidth	2.3 GHz
Target’s stance	Sitting on a chair
Power consumption	120 mW
Mean output power	55 μW
Peak-to-peak output amplitude	0.69 V

**Table 2:** SNR of respiration and heartbeat signal using FIR filter and the proposed method.

Parameters	FIR	Proposed Method
Respiration SNR	4.44 dB	12.03 dB
Heartbeat SNR	−53.52 dB	−48.70 dB
